# Yield estimation of the 2020 Beirut explosion using open access waveform and remote sensing data

**DOI:** 10.1038/s41598-021-93690-y

**Published:** 2021-07-08

**Authors:** Christoph Pilger, Peter Gaebler, Patrick Hupe, Andre C. Kalia, Felix M. Schneider, Andreas Steinberg, Henriette Sudhaus, Lars Ceranna

**Affiliations:** 1 Federal Institute for Geosciences and Natural Resources (BGR), 30655 Hanover, Germany; 2 GFZ German Research Centre for Geosciences, 14473 Potsdam, Germany; 3 Christian Albrechts University, 24118 Kiel, Germany

**Keywords:** Geophysics, Seismology, Acoustics, Imaging techniques, Atmospheric dynamics

## Abstract

We report on a multi-technique analysis using publicly available data for investigating the huge, accidental explosion that struck the city of Beirut, Lebanon, on August 4, 2020. Its devastating shock wave led to thousands of injured with more than two hundred fatalities and caused immense damage to buildings and infrastructure. Our combined analysis of seismological, hydroacoustic, infrasonic and radar remote sensing data allows us to characterize the source as well as to estimate the explosive yield. The latter is determined within 0.13 to 2 kt TNT (kilotons of trinitrotoluene). This range is plausible given the reported 2.75 kt of ammonium nitrate as explosive source. As there are strict limitations for an on-site analysis of this catastrophic explosion, our presented approach based on data from open accessible global station networks and satellite missions is of high scientific and social relevance that furthermore is transferable to other explosions.

## Introduction

The explosion that occurred in the city of Beirut, Lebanon, on the 4th of August 2020 around 18:08 local time (15:08 UTC) was caused by the combustion of approximately 2.75 kt of ammonium nitrate stored in a harbour warehouse, as announced by the government shortly afterwards. This accident led to thousands of casualties with more than two hundred fatalities^1^. An enormous shock wave following the explosion caused immense damage to buildings and infrastructure, also shattering windows all over the city. On-site investigations into the cause and nature of the explosion were conducted by local authorities^2^. Nevertheless, access to an explosion site can be limited for various reasons. For instance, approaching the site can be harmful when chemicals or radioactivity pollute the area. Consequently, on-site information and data can be sparse, or there is a need for an independent validation. For the explosion in Beirut, some of those limitations were in place, also due to its timing in the SARS-CoV-2 pandemic. So for significant events with a large impact on people, like in the case of Beirut, transparent investigations that use open methods and publicly available data to analyse explosions reliably are important. We therefore offer an independent, third-party estimation of the explosive yield from the analysis of publicly available waveform and remote sensing data. All yield estimates are provided in kilotons of trinitrotoluene (TNT) equivalent (the formal definition of 1 kt of TNT is a trillion ($10^{12}$) calories).

During the last 100 years a huge number of various anthropogenic explosions were remotely detected by seismic or acoustic sensors. The first explosion that was investigated in order to study acoustic wave propagation in the atmosphere occurred in 1921 at a BASF plant in Oppau, Germany^3^, also originating from the combustion of ammonium nitrate. Subsequently, many man-made explosions were recorded during the era of nuclear testing (1945 to 1998)^4^ until the Comprehensive Nuclear-Test-Ban Treaty (CTBT) was opened for signature^5^. In later years, only the Democratic People’s Republic of Korea performed (underground) nuclear tests, which were consistently detected by CTBT’s International Monitoring System (IMS) and other seismic and infrasonic stations as well as satellite remote sensing^6,7^.

The IMS as well as other seismic and infrasonic networks recorded a large number of further accidental explosions during the 21th century^8–11^. Albeit the catastrophic nature of many of these explosions, such events provide valuable datasets for remote sensing and propagation studies as well as monitoring and verification issues.

The sub-audible sound waves (i.e. below 20 Hz) of the Beirut explosion propagated through the atmosphere to distances of thousands of kilometers and were recorded by infrasound arrays of the IMS. Seismic, hydroacoustic and acoustic signals propagated through solid earth, water and air, respectively, and were recorded at nearby land-based and ocean-bottom seismometers, which we present and exploit for our analysis in this paper. Furthermore, we infer damages to buildings in the city with spaceborne synthetic aperture radar (SAR) imagery from before and after the explosion and link damage maps with overpressure caused by the explosion. Here we analyse this combined dataset within the present study to benchmark origin time and epicentre of the event. We focus on a consistent yield estimate based on results from the different methods.

**Figure 1:**
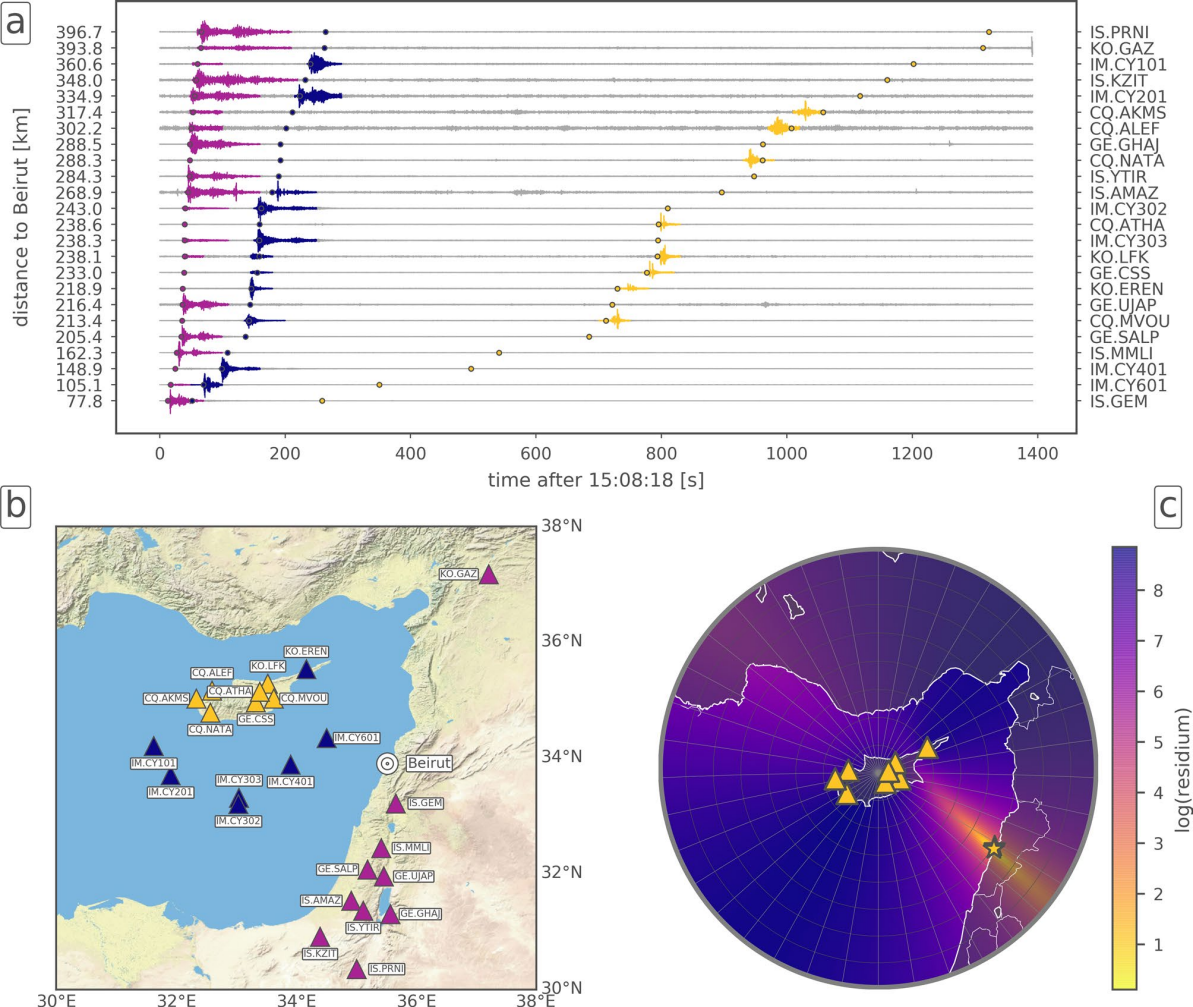
Data from regional seismic stations. (**a**) Waveforms of the seismic stations. Colored circles (purple, blue, yellow) represent theoretical arrivals of phases propagating with seismic (6.0 km/s), hydroacoustic (1.5 km/s) or acoustic (0.3 km/s) velocities. Observed waveform arrivals of seismic, hydroacoustic or acoustic phases are color coded in the respective colors. Waveforms are bandpass-filtered in the frequency band 0.5 to 8.0 Hz. (**b**) Seismic stations with distances of up to 400 km from the explosion site in Beirut. Station colors represent the type of the dominant observed phase (seismic = purple, hydroacoustic = blue, acoustic = yellow), see also subfigure (**a**). (**c**) Localization of the event based on acoustic phases (marked yellow in subfigure (**a**)) detected on the eight seismic stations at the island of Cyprus for details). Background colors represent a residuum, which defines the optimal localization at its smallest value. The best localization is found at 33.863$^{\circ }$ N, 35.502$^{\circ }$ E, marked as yellow star at Beirut (see “Seismoacoustic localization procedure for stations located on Cyprus” section for details on the localization procedure). Maps in subfigures (**b**) and (**c**) are created using the Matplotlib Basemap Toolkit (https://matplotlib.org/basemap/, last access: 2021/02/15). Background topography in subfigure (b) is available from the Esri ArcGIS Map Service; country and coastline outlines used in subfigure (**c**) are provided by the Global Self-consistent, Hierarchical, High-resolution Geography Database (GSHHG) and by the Generic Mapping Tool (GMT), respectively.

## Waves from the explosion traveling through Earth, ocean and atmosphere

We inspected publicly available data from seismometers located in the region around Beirut. Multiple signals in distances of up to 400 km have been detected (see Fig. 1a), where energy propagated as seismic, hydroacoustic and acoustic waves through ground, ocean and atmosphere, respectively. The map in Fig. 1b shows the spatial distribution of the stations color coded by the dominant phase detected. Figure 1c shows the localization of the event based on seismoacoustic phases recorded at the seismometer network in Cyprus (see below).

On seismometers north and south of Beirut the seismic phases are dominant. These have been observed on the majority of the inspected stations. Due to the location of the explosion in the harbour of Beirut, part of the energy has also been released directly into the water, which caused the hydroacoustic signal to be the dominant phase on all ocean bottom seismometers (station codes beginning with IM.CY), located in the eastern Mediterranean Sea south of Cyprus. Moreover, by hydroacoustic-to-seismic coupling this signal was also clearly recorded on island stations located near the coast of Cyprus (CQ.MVOU and KO.EREN).

## Event localization and characterization by seismoacoustic and seismic phases

Being located in north-western directions at distances of around 200 to 300 km, Cyprus is situated in a favourable area for seismoacoustic observations from Beirut during summer. All seismometer stations located on Cyprus detected acoustic signals that have been coupled into the ground by acoustic-to-seismic coupling^12^, as observed also at other large explosions^10^. Infrasound most efficiently propagates from the source to receivers in a stratospheric waveguide^13^, in which the signal energy is continuously reflected between the surface and the upper stratosphere while the damping is low. This waveguide evolves if the along-path wind speed in about 50 km is sufficiently high such that the effective sound speed equals or exceeds the sound speed at the ground; i. e. the effective sound speed ratio ($v_{\mathrm {eff-ratio}}$) equals or exceeds one. In the summer, the easterly stratospheric winds favor infrasound detections in western direction. As a consequence of upward refraction from a source at the surface followed by downward refraction from stratospheric altitudes, acoustic signals are not detectable in an acoustic shadow zone regularly establishing up to 200 km. Thus, the observation ranges for seismoacoustic phases are limited in distance and azimuth depending on the atmospheric conditions. In the area of the first surface bounces, the acoustic energy is largest and most likely detectable by seismometers. Using the seismoacoustic phases from seismic stations on Cyprus, we localized the origin of the explosion. The yellow star in Fig. 1c marks the best location, which we found at 4.8 km south of the harbour of Beirut. Our localization technique confirms the acoustic nature of the signals (apparent velocity of 344 m/s) as well as the source origin (see “Seismoacoustic localization procedure for stations located on Cyprus” section for details).

We invert the observed regional broadband seismic waveforms recorded in distances up to 400 km to characterize the explosion in terms of onset time, duration and strength. Also our ability to well locate the explosion is tested here, even though the location is known. For representing the explosion we use a volumetric source at the surface (“Explosion source inversion” section). Our estimates give an onset time for the explosion of 15:08:18.63 UTC, a duration of 2.9 s and a location at 33.91$^{\circ }$ N, 35.52$^{\circ }$ E. The moment tensor representation is estimated to relate to a seismic moment magnitude $M_w$ 3.47. We can now relate the seismic moment via the shear stress change to the energy of the explosion [“Yield estimation from seismic data” section (1)], which results in a yield estimate of 1.08 kt.

We apply an additional method to estimate the yield of the explosion from seismic data. This approach relies on the relation of teleseismic body wave magnitude $m_b$ measurements to the seismic yield of an explosion [“Yield estimation from seismic data” section (2)] and results in an estimate of 0.13 to 0.34  kt TNT for the explosion. These values have to be considered a lower bound estimate, as the relations are established for well-coupled underground nuclear explosions. For a surface explosion only a small portion of the total energy couples into the subsurface^14^ as seismic energy and is subsequently considered in the $m_b$ measurements. For the estimation based on the body wave magnitude we further assume that the explosive conversion from chemical energy takes place simultaneously resulting in an instantaneous release of seismic energy.

## Infrasonic signatures observed at thousands of kilometers distance

We analyse data from IMS infrasound arrays in distances up to 10,000 km to identify signatures potentially related to the Beirut explosion. We apply the progressive multi-channel correlation (PMCC) method^15^ (see “Infrasound array analysis, location and yield estimation” section). The back-azimuths, the apparent velocities, and the onset times at the different arrays enable estimating the event location, and we use peak-to-peak amplitudes as well as dominant periods for yield estimations.

**Figure 2:**
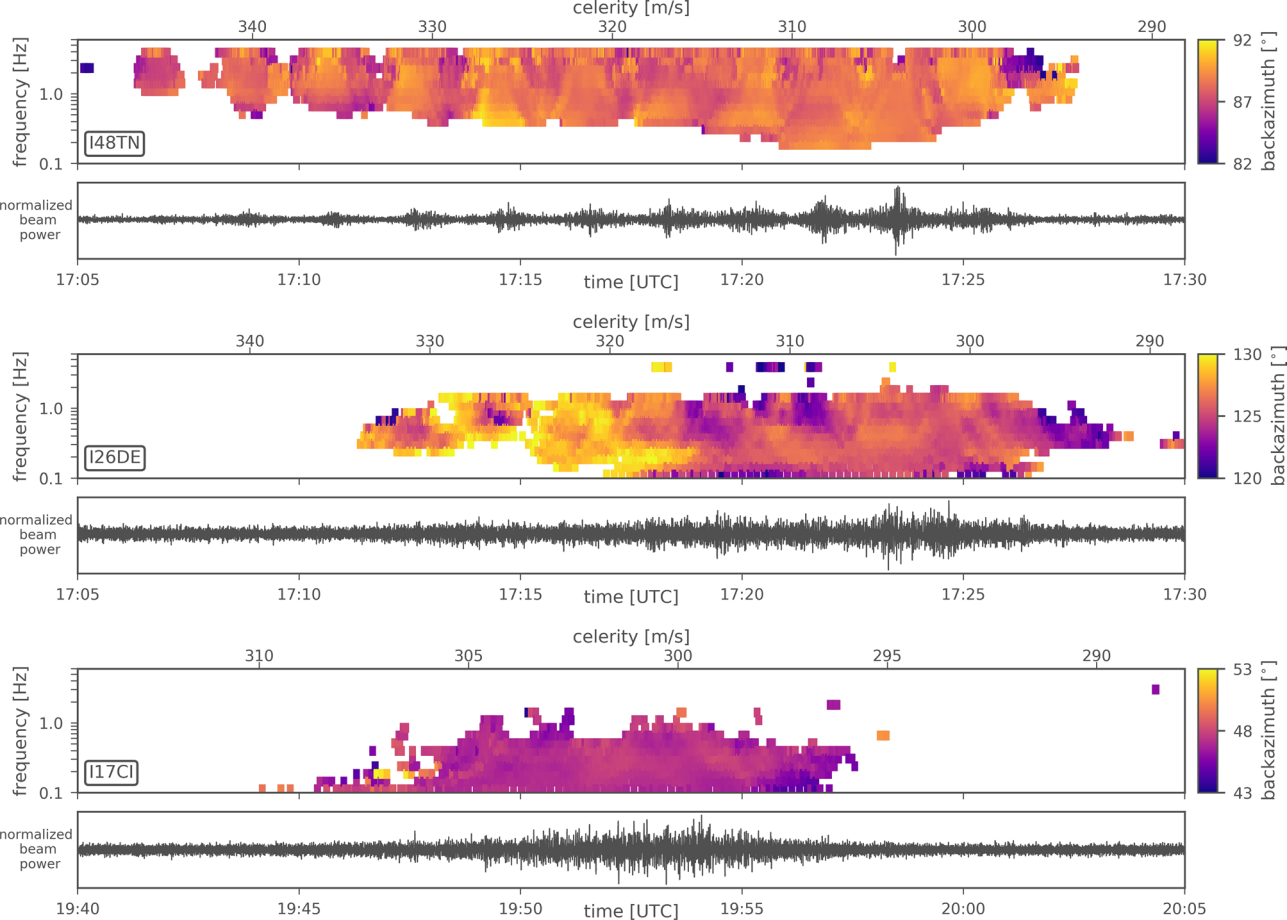
Infrasound analyses for the three stations I48TN in Tunisia, I26DE in Germany and I17CI in Ivory Coast using the PMCC method. Top frames show color-coded back-azimuth information of each time-frequency-pixel in a 10$^{\circ }$ segment centered around the true direction towards Beirut. Bottom frames show corresponding waveform beams of differential pressure, bandpass-filtered between 0.5 and 8.0 Hz.

The array analysis yields detections associated with the explosion at five IMS infrasound stations (see Table 1): I48TN (Tunisia), I26DE (Germany), I17CI (Ivory Coast), I42PT (Azores) and I11CV (Cape Verde Islands). We focus on I48TN, I26DE and I17CI as these exhibit excellent infrasound records with the highest signal content (see Table 1) and thus lower parameter uncertainty. Figure 2 shows their PMCC analyses. A remarkable feature visible in the station recordings is the signal separation into various pressure pulses at I48TN. These pulses are related to the separation of signal energy of one single blast into multiple stratospheric propagation paths from the source to the receiver, as was also observed for the Buncefield explosion in 2005^8^.

**Figure 3:**
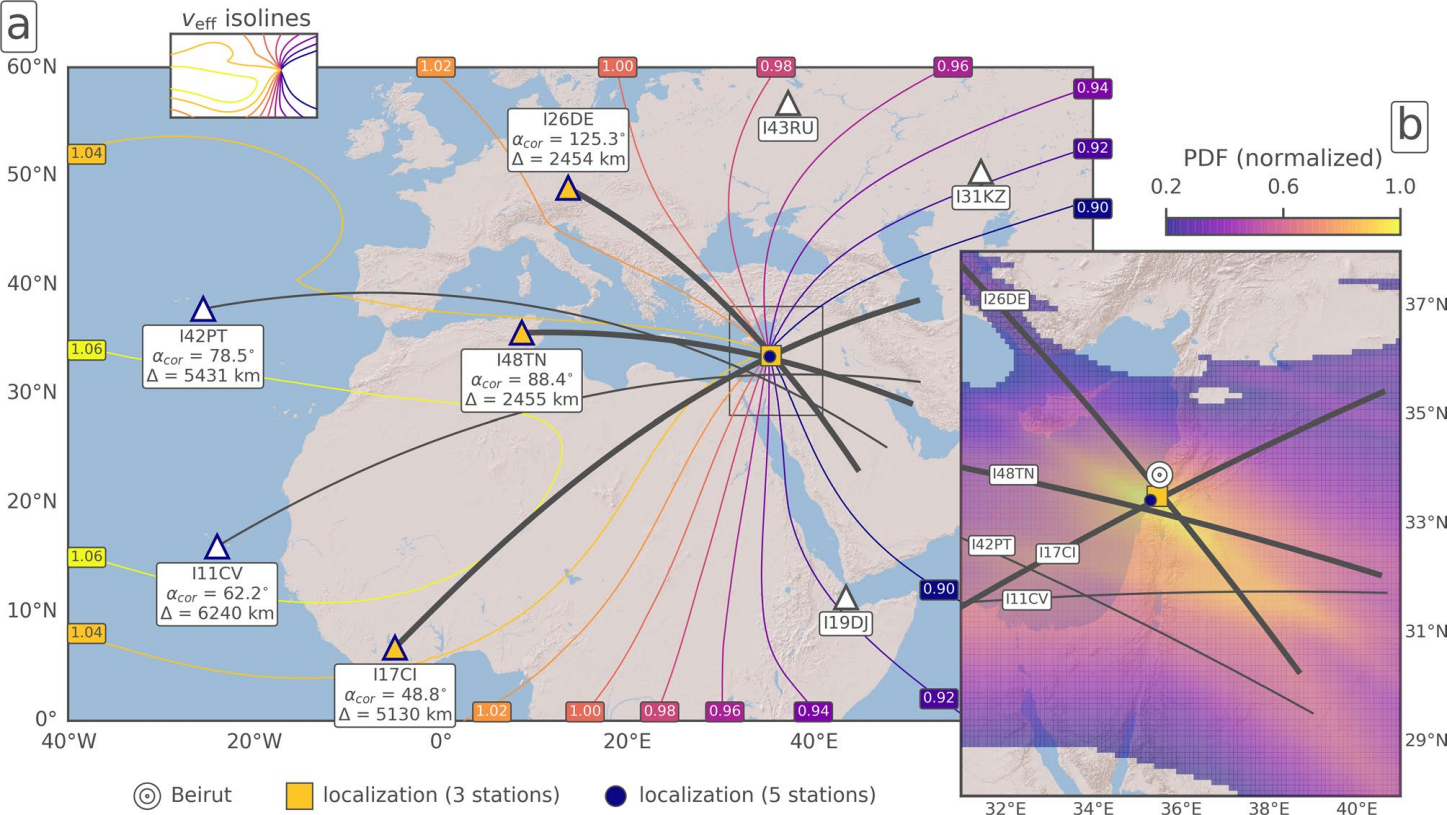
Localization of the explosion using IMS infrasound data. (**a**) IMS stations (triangles) and effective sound speed ratio ($v_{\mathrm {eff-ratio}}$) isolines respective to the source location, based on the HWM14 model for 16:00 UTC. The great-circle projections (black lines) correspond to the ECMWF-corrected PMCC back-azimuths ($\alpha _{cor}$) of the detecting stations. The stations with the highest signal content—given in Fig. 2 and used for yield estimation—are highlighted (yellow triangle, thick lines). IMS stations at which $v_{\mathrm {eff-ratio}}<0.98$ did not detect the explosion due to the prevailing easterly stratospheric winds. (**b**) Details of the localization using the grid-search approach (see “Infrasound array analysis, location and yield estimation” section). The maximum of the normalized probability density function (PDF) depicts the optimum location of the grid-search approach using the five detecting stations. It results in a deviation of 56 km to the southwest (blue circle) of the actual origin (white circle). Relying on the three best-detecting stations results in a slightly improved location at 44 km to the south (yellow square) as the back-azimuth uncertainty is smaller. Maps are created using the Matplotlib Basemap Toolkit (https://matplotlib.org/basemap/, last access: 2021/02/15). Background topography is available from the Esri ArcGIS Map Service.

Figure 3 shows the IMS infrasound stations detecting the Beirut explosion as well as the propagation conditions depicted by $v_{\mathrm {eff-ratio}}$ (a) and the localization results (b). The source location (see “Infrasound array analysis, location and yield estimation” section) that we determine using five IMS stations is 56 km south (33.4334$^{\circ }$ N, 35.3067$^{\circ }$ E) of the actual origin; whereas we improve the localization (33.5067$^{\circ }$ N, 35.4666$^{\circ }$ E) to only 44 km south of the actual origin when we use only the three best detecting stations. Given the large station distances, this result proves the capability of the IMS infrasound network for event localization.

We estimate the explosive yield based on data of the three infrasound arrays I48TN, I26DE and I17CI. We apply the AFTAC and LANL relations (see “Infrasound array analysis, location and yield estimation” section), the latter one using both the climatological and Horizontal Wind Model as of 2014 (HWM14)^16^ and the high-resolution analysis of the European Centre for Medium-range Weather Forecasts (ECMWF). The input parameters and resulting yields are summarized in Table 2 with final explosive yield estimates derived by averaging over the three stations.

In general, infrasound-based yield relations, including those of LANL and AFTAC, are associated with large uncertainties, which can amount from a factor of two up to more than one order of magnitude^17^. We find that the AFTAC and LANL (HWM14) relations correspond well in resulting maximum yields around 1 kt TNT but differ in range (0.86–1.47 kt TNT opposed to 0.22–1.35 kt TNT). Although the AFTAC relation is primarily used for explosive sources at higher altitude like meteors^18^ and is independent of the actual ducting conditions, it is applicable here because we only consider stations within good stratospheric ducting conditions. The LANL relation is highly sensitive to the atmospheric dynamics since the measured amplitude can be affected by station-dependent noise conditions and its wind correction depends on the accuracy of models representing stratospheric altitudes. Related uncertainties are likely a cause for the large spread of more than 1 kt TNT between the stations using the climatological HWM14 model. For comparison, we account for more precise ECMWF profiles in the LANL relation we estimate a yield of about 0.5 kt TNT with a similarly large spread (0.14–1.12 kt TNT). However, the empirical LANL relation is based on wind speeds derived from climatologies. Therefore, precise ECMWF profiles seem to be less appropriate for estimating yields with the LANL relation and will not be considered any further.

## Linking overpressure simulations and InSAR derived damage proxy maps

**Figure 4:**
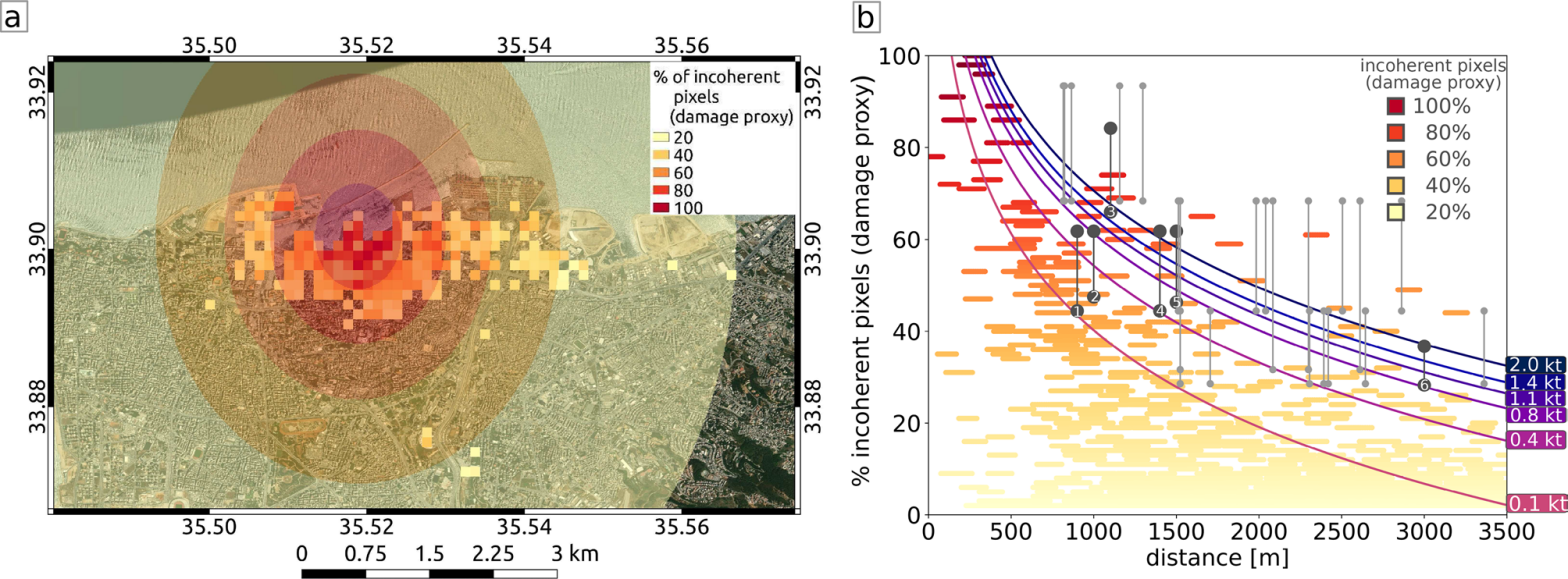
Yield estimation through comparison of approximated and estimated damage over distance from the explosion site. (**a**) Damage proxy (colored pixel) and expected damage map over an optical satellite image background (Sentinel-2). The damage proxy is percent of pixels that experienced significant loss of interferometric coherence after the explosion in a windowed damage pixel of 100 m by 100 m size. The transparent overlay indicates the radii of the expected damage from a yield of 0.8 kt TNT and is colored after the relative damage classes^22^. Map background was created using basemap data from the Esri ArcGIS Map Service. (**b**) Measured damage and expected damage for several evaluated yields over distance from the explosion site. “Yield estimation from InSAR damage maps” section Eq. 3 is evaluated for yields of 0.1, 0.4, 0.8, 1.1, 1.4 and 2.0 kt of TNT at the distances of the damage pixels and plotted as lines. Damage proxy is plotted as in (**a**). Vertical lines mark damage proxy estimations for reported damage which we relate to peak overpressure at locations given in Table 4 (black, with numbers) and from a dedicated damage survey^23,24^ (gray).

The blast of the explosion caused a wide range of damages to buildings. The reflection of radar waves from SAR satellites is strongly depending on the ground structure down to a decimeter level, with a sensitivity to less than a centimeter of motion. In areas of Beirut where the outsides of buildings have been significantly damaged, space-borne SAR images showing the backscattered amplitude and phase of radar waves before and after the explosion therefore differ. To quantify this difference and thereby approximating the building damage, we use a combined measure of the amplitude and phase similarity in multitemporal SAR image pixels, the interferometric coherence, and we relate changes in this measure to damage (Fig. 4a, “InSAR damage maps” section). For image pairs of two ascending and two descending InSAR tracks (Table 3) we calculate the interferometric phase coherence using a 5 by 5 pixel window between acquisitions from before and after the explosion, and derive four coherence change maps. A large drop of coherence may occur when e.g. buildings collapse, while a small coherence drop can be caused by e.g. damages of building facades. The size of the estimation window used for coherence estimation is a trade-off between overestimation of coherence for small window sizes^19^ vs. spatial resolution for large window sizes. In this study, an estimation window of 5 x 5 pixels was applied on 20 x 5 m Sentinel-1 data, resulting in a 100 x 25 m coherence estimation window. The phase coherence has a maximum value of one and we assume that a coherence loss of $\ge$0.2 is significant and indicates change in the radar wave reflection due areas characterized by damage of vulnerable surfaces of man-made infrastructure. In our coherence loss estimation we combine the findings from all four coherence change maps (“InSAR damage maps” section): if a pixel in any of the four coherence maps shows significant coherence loss, we assign a damage pixel in the combined binary image. Further we consider a high density of damage pixels in form of area percentage is a proxy for high damage to individual structures in that area. We calculate the percentage of damage pixels in a 10 by 10 pixel window (roughly 100 by 100 m), which forms the damage proxy map (Fig. 4a). Despite the relatively short time interval between all analyzed SAR acquisitions, coherence loss can have various other sources, such as ever changing vegetation and mobile larger objects, e.g. ships and ship containers. A high density of coherence loss, especially close to the explosion site in a city, however, is likely pointing to widespread structural changes and therefore damage caused by the explosion. Our damage proxy is strongly biased by the density of vulnerable infrastructure. Localized strong damage could be washed-out where vulnerable infrastructure is missing in the surroundings. Next we calculate the explosive yield necessary to produce such damage. Most damage of an explosion is caused by the produced overpressure. Joint inversion of overpressure data and seismological data has been done to robustly estimate the yield in previous work^20^, showing the usefulness of consideration of overpressure data. The empirical relation “BOOM”^21^ developed from conventional explosive tests between 0.1 and 1 kt of TNT is used to relate the yield of an explosive to its resulting peak overpressure *P* (in Pa) at any given distance *r* (in m) for a surface explosion. We assume a relation between peak overpressure and resulting damage^22^. Damage to structures caused by the external force of an explosion will first act on the external surface of affected structures. We think it is therefore reasonable to assume a relation between the density of the with InSAR data observed changes to surfaces in an area, as described by the damage proxy, and the explosion force causing overpressure-related damage to structures. We set 80 kPa peak overpressure to result in 100% of damaged surface, the damage proxy, at all structures. Widespread dense damage to buildings likely requires high peak overpressure, while less dense damage not necessarily points to lower peak overpressure values. The wide range of different damage proxy values at a certain distance is likely caused by variable density of vulnerable surfaces and variable pressure wave attenuation due to variable diffraction in the surrounding of the explosion. We use ground-truth available from media reports (Table 4) and an openly available report of on-site damage inspections of buildings throughout the city^23,24^ to calibrate between our pixel damage proxy and the damage as a function of peak overpressure^22^. This on-site damage report^23,24^ categorizes four damage classes which we relate to peak overpressure ranges as follows: class 1) 1 to 3.5 kPa, class 2) 3.5 to 7 kPa, class 3) 7 to 20 kPa and class 4) 20 to 60 kPa. The resulting pixel damage proxy is calculated according to “Yield estimation from InSAR damage maps” section Eq. 3 and shown in Fig. 4b. We evaluate the developed relation of peak overpressure with distance to the explosion and the mapped damage proxy from InSAR data in comparison with the necessary yield in TNT.

The scaled empirical overpressure-damage function in Fig. 4b for a yield of 1.1 kt TNT traces the upper maximum values of the damage proxy for distances smaller than 1000 m. Below 1000 m distance from the explosion, damage values higher than 40% are bound by a minimum yield of 0.4 kt TNT (Fig. 4b). We think that the explanation of the damage close to the explosion source is most important, as the overpressure will cause damage to the structures without attenuation or focusing effects. Also, we expect different vulnerabilities of individual structures possibly become more significant for lower overpressure values at greater distances. The damage close to the explosion source is best explained by a yield of 0.8-1.1 kt TNT. The expected spatial distribution of damage proxy values for a yield estimate of 0.8 kt TNT are shown in Fig. 4a. The damage at the Seaside arena located roughly at 1 km distance across the harbour requires , based on our relation, a minimum explosion yield of 0.75 kt TNT to be well explained. Beyond distances of 1000 m from the explosion the expected damages from the peak overpressure damage relation fall off stronger than some of the InSAR damage proxy values and damage class estimations at inspected damaged buildings^24^. At these larger distances also larger yields around 2 kt TNT may well explain the high damage proxy values.

## Discussion

The Beirut 2020 explosion coupled into ground, water and air and accordingly generated seismic, hydroacoustic and infrasonic wave signatures observed and analysed within this study. We inferred the temporal and spatial source characteristics using seismic and acoustic methods, resulting in a location accuracy of 44 km using remote IMS infrasound arrays and below 1 km when using regional seismic stations.

All three independent methods that we applied (seismological analysis, acoustic yield relations, satellite radar image analysis) consistently estimate the range of the yield within one order of magnitude. The lowest yield estimate of 0.13 kt TNT is given by the seismic body wave analysis and the largest yield estimate of up to 2 kt TNT stems from relating reported damage and an InSAR damage proxy to peak overpressure. It has been shown that such yield estimates, even under ideal conditions and especially for chemical explosions, can vary strongly^20^. For this study we believe that methodological uncertainties such as energy coupling from the surface into the ground or atmospheric propagation modelling are the most significant contributors to uncertainties in our yield estimations.

Recent studies^25–27^ based on the analysis of video images calculate yield estimates in the range from 0.3 to 1.12 kt TNT equivalent, with the upper bound being reported as an upper limit estimate^25^. These estimates based on completely different approaches support our findings. At close range ($<\,1000\,\text {m}$) the InSAR damage proxy relation to peak overpressure confines the upper yield limit to 1.1 kt TNT equivalent. Overall the InSAR damage proxy relation to peak overpressure relation under our assumptions gives a yield estimate of 0.4 to 2 kt TNT equivalent. The seismic body wave yield estimate of 0.13 to 0.34 kt TNT serves as a lower bound with only partial energy coupling into the subsurface. With an announced amount of 2.75 kt of ammonium nitrate as the source of the explosion and considering that ammonium nitrate has an explosive efficiency of about 50% of TNT, a yield of about 1.4 kt TNT equivalent can be expected. This is consistent with our yield range estimates of 0.13 to 2 kt TNT equivalent.

We developed a novel approach to infer the yield of an explosion from damage proxies inferred by changes in radar satellite remote sensing images from before and after the explosion. Most damage to structures due to an explosion is caused by overpressure. Each explosive yield results in a specific overpressure level at given distances. This allows us to estimate the yield of the explosion by relating the expected radius and expected damage to a damage proxy derived from observed changes in radar satellite remote sensing images. This approach is operationally feasible because of the unique Sentinel-1 mission characteristics. They include the long-term mission design, a continuous acquisition plan of the Earth’s surface with high frequency and a free, full and open data policy.

Considering the design goal of the CTBT IMS network to detect any explosion of at least 1 kt TNT equivalent, we were able to reliably identify, locate and characterize an atmospheric (surface) explosion around that magnitude with three (and more) infrasound stations. Nevertheless, improvements of the location accuracy and an independent mean to confirm the origin and yield of an explosion can be provided by considering freely available seismometer and spaceborne remote sensing data as national technical means to the monitoring and verification capabilities of the CTBT.

## Methods

### Seismoacoustic localization procedure for stations located on Cyprus

**Figure 5:**
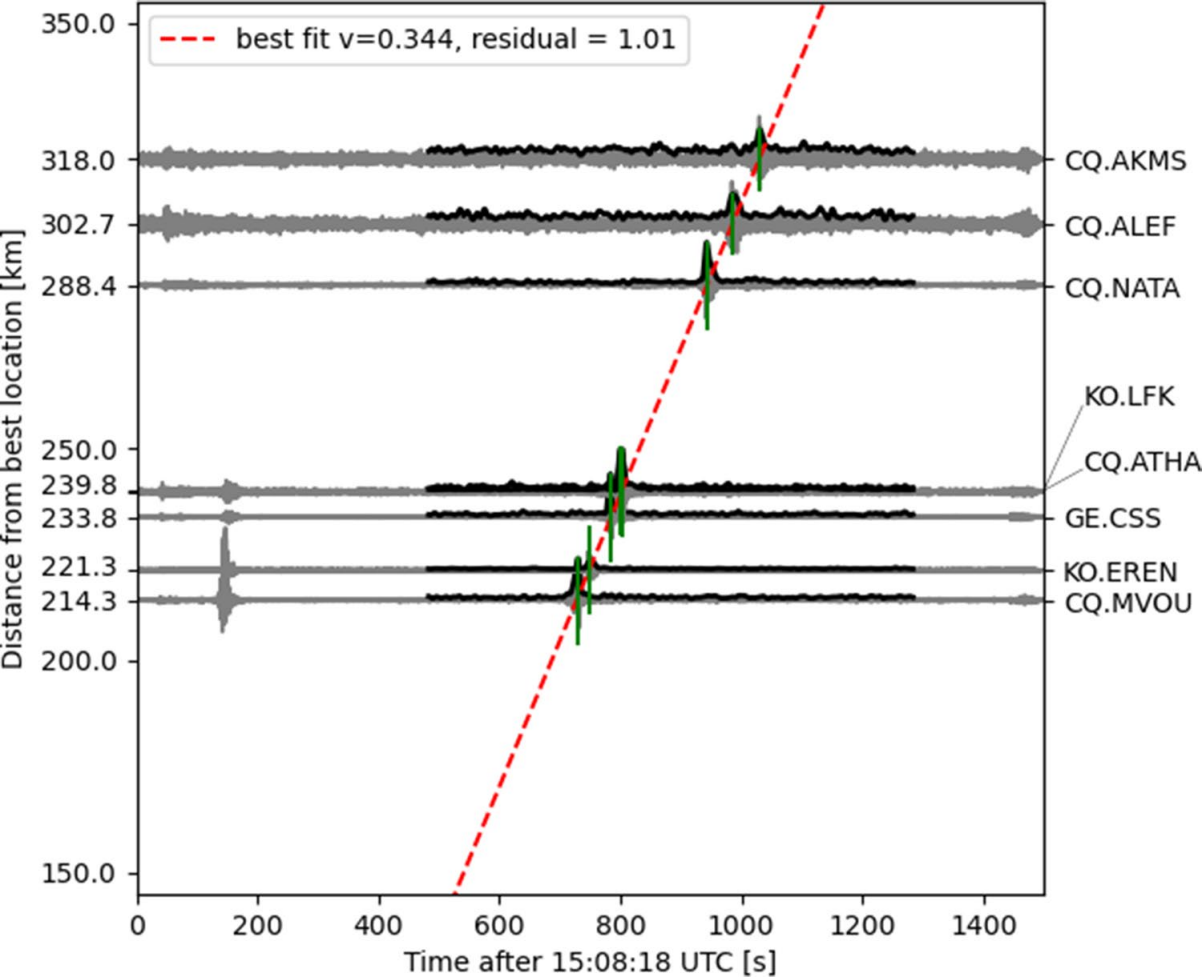
Seismoacoustic localization procedure for seismic stations located on Cyprus. The figure shows continuous waveforms (grey) and their smoothed envelopes (black) in the selected time window. The maxima of the latter were used to define the arrival times (marked as green lines). During grid-search, for each location the least-squares solution is derived, that best fits a straight line through the arrival times. Here, the case of the lowest residuum is shown, which is achieved for an apparent velocity $v=0.344$ km/s at the location (33.863$^{\circ }$ N, 35.502$^{\circ }$ E) (see yellow star in Fig. 1c).

We performed a grid-search to localize the source of the acoustic signals detected at a network of eight permanent seismic stations on Cyprus. We adapted conventional array analysis to the given situation of a local network which is situated in a restricted azimuth and distance range on the one hand, but which has, on the other hand, a too large aperture to assume plane wave propagation across the network, as commonly applied for beam forming on small scale arrays. Therefore our grid-search evaluates the consistency of the observed apparent velocities taking into account spherical wave propagation from each test location. Figure 1c shows the result for distances ranging from 0 to 400 km and back-azimuths ranging from 0$^{\circ }$ to 360$^{\circ }$ with distance and back-azimuth spacings of 2 km and 1$^{\circ }$, respectively, with respect to the center location of the Cyprian network. For each location, we performed a linear regression analysis in order to find the apparent velocity fitting the observed seismoacoustic arrivals best. The residuum of the least-squares solution is color coded logarithmically as background colors in Fig. 1c. The lowest residuum defines the best localization, which is found at (33.863$^{\circ }$ N, 35.502$^{\circ }$ E), marked as yellow star. This localization is located 4.8 km south of the ground truth location at the harbour of Beirut. We used an automatic procedure to define the arrival times of the acoustic phase at the eight seismic stations at Cyprus. The *z*-components of each station were investigated in a time window of ±400 s with respect to a rough arrival time estimate, supposing a celerity of 0.3 km/s and taking into account the average distance from Beirut. Waveforms were bandpass-filtered between 0.5 Hz and 4 Hz and normalized to their maximum amplitudes. In order to get rid of waveform variations due to different atmospheric paths we used smoothed envelopes of the traces. We defined the maxima of the smoothed envelopes as arrival times. Smoothing was done by a zero-phase Butterworth lowpass filter with a corner period of 10 s. Figure 5 shows the original seismic traces (gray), the smoothed envelopes and the resulting straight line (red dashed line), which fits best the arrival times at the location with the minimum residuum. The vertical offsets of the traces are proportional to the distance of the respective stations from the best localization. Note that the resulting velocities are not absolute velocities (celerities) but an average trace velocity across the network. Thus the resulting velocity cannot be used to infer the origin time. However, by assuming an average celerity of 0.3 km/s, the origin time can be inferred for the best localization as 15:08:13.4 UTC ± 6 s. This estimation is derived by subtracting the theoretical travel times from the absolute arrival times at the individual stations. The error is defined by the standard error of the mean. The result is in agreement with the ground truth time of 15:08:18 UTC.

### Explosion source inversion

We carry out an inversion of regional broadband seismic waveforms up to 400 km distance to infer source parameters. We assume an explosion source with the source parameters location (lat, lon), time, duration, depth and magnitude. We fix the depth at the surface, as a result we forward model a pure vertical force). We allow the location to vary around 1 km from the known location of the blast. We use the “Grond” optimization algorithm^28^. Synthetic waveforms are calculated using a 20 Hz Green’s function store using the QSEIS code by Wang (1999)^29^ and based on a composite 1-D velocity model^30,31^. We downsample the waveform records to 20 Hz. We filter with a Butterworth bandpass of fourth order between 1.2 and 3 Hz for stations up to 90 km distance and between 3 and 4 Hz for stations farther away. We compare synthetics and data in a tapered window between 0.5 s before and after the theoretical P-wave onset for the closest station and between 0.5 s before and 2.5 s for all other stations. To compensate for unmodeled 3-D path effects we allow at each station an individual shift of the trace of up to 1 s to maximize fit. We assign the closest station manually double the weight in comparison to other stations. We make the results available online as a report under https://www.seismologie.bgr.de/sdac/erdbeben/big_quakes/beirut_report/index.html#/. We note that the choice of the velocity model and especially the choice of the top layer, in which the explosion occurs, influences the yield estimate. Without knowledge of the onsite soil and shallow sub-surface structure a choice of strong strong consolidated sediment as assumed in the employed velocity model seems reasonable to represent a upper limit of the rheological parameters. Weaker soil structures will lead to a higher yield estimate. Our choice reflects the capability of the seismological data to infer a lower bound estimate of yield estimate as also the seismic coupling factor is unknown. We find the best fitting source parameters to be a magnitude of 3.47 (seismic moment of $1.8\times 10^{14}$ N m after Hanks and Kanamori^32^) with a source duration of 2.9 s and an onset time of source time to be 15:08:18.63 UTC and the origin location to be 33.9050$^{\circ }$ N and 35.5185$^{\circ }$ E. The position of the source can be well constrained in the north direction (standard deviation $< 0.1\,\text {km}$), but not the east direction, with a standard deviation of  0.6 km. This is a result of the station distribution lacking stations in the east.  70% of the observations can be explained by the forward model.

### Yield estimation from seismic data

Two methods are applied to estimate the yield of the explosion using seismic data.

1. The first method to estimate the yield of the explosion relies on the relation of seismic moment and strain energy drop via the shear stress change $\Delta \sigma$ and shear modulus $\mu$^33^:
  1 $$\begin{aligned} E = \frac{\Delta \sigma }{2 \mu } M_{0} \end{aligned}$$
  This relation is developed for for a shear-crack and will result in an lower limit estimate of the released energy for a surface explosion, as non-adiabatic processes are not taken into account. Evaluating “Yield estimation from InSAR damage maps” section Eq. 2 for a yield of 1.1 kt TNT very close to the source (0.01 m distance) leads to roughly $10^{8}$ Pa stress change. Assuming the seismic moment $M_0$ of $1.8\times 10^{14}$ Nm determined in “Explosion source inversion” section and the $2\times 10^{9}$ Pa shear modulus based on the used velocity model results in a yield estimate of 1.08 kt TNT. This approach implicitly assumes the yield for estimating the shear stress change from “Yield estimation from InSAR damage maps” section Eq. 2. This nevertheless validates the consistency between “Yield estimation from InSAR damage maps” section Eq. 2 and the determined seismic moment. We note that the empirical relations and assumptions of “Yield estimation from InSAR damage maps” section Eq. 2 are probably invalid very close to the source and that the choice of the distance evaluated strongly impacts the resulting yield estimate. Relations between energy and seismic moment are not straightforward and the assumption is taken that the shear stress change can be estimated at first order from the pressure change, which neglects other energy conversion contributions during an explosion.
2. The second method of yield estimation applied here relies on the relation of seismic body wave magnitude ($m_b$) measurements to the yield of an explosion. This relation is commonly and widely used in the field of nuclear underground test monitoring, but might also be used to provide a lower bound of an explosive source on the surface. The relation between $m_b$ and yield depends on multiple factors, such as the geological setting at the source site, the efficiency of wave propagation from source to receiver, the depth of the explosion, as well as the coupling of the source to the underground. Due to these factors it is difficult to state one single relation, but rather empirical relations developed for different areas are required. These empirical formulas are of the type $m_b=A+B\,\mathrm {log(}Y\mathrm {)}$, where *Y* is the seismic yield of the explosion in kt TNT equivalent and *A* and *B* are constants depending on the aforementioned factors. These empirical equations have been used in different regions for calibration, for example at the Nevada test site^34^ ($Y=3.92+0.81\,\mathrm {log(}Y\mathrm {)}$), in Kazakhstan^35^ ($Y=4.45+0.75\,\mathrm {log(}Y\mathrm {)}$) or in Nova Zemlya^36^ ($4.25+0.75\,\mathrm {log(}Y\mathrm {)}$). As these relations only hold true for well coupled underground explosions, seismic coupling factors for above ground explosions given by Bornmann et al. (2009)^14^ have to be taken into account. The International Data Center (IDC) of the CTBT organization states a body wave magnitude ($m_b$) of 3.2 for the Beirut explosion in its Reviewed Event Bulletin. This value for $m_b$ can be related to seismic yield in kt TNT equivalent of the explosion. According to Brax et al. (2016)^37^ the geological unit at the explosion site is comprised out of dolomite rocks and the region can be classified as International Building Code (IBC) class C (very dense soil and soft rock). Under these geological assumptions we use $m_b$ yield relations for wet hard rock^34^ and for dry unconsolidated rock^7^ to estimate a range of explosive yield of the explosions. These relations result in a yield estimate of 0.13 to 0.34 kt TNT equivalent for the explosion. For surface explosions only a small portion of the total released energy couples into the subsurface as seismic energy. According to Bornmann et al. (2009)^14^ seismic coupling factors for a surface explosion can be as low as 0.1% and therefore our yield assessment based on $m_b$ relations has to be considered as a lower bound estimate.

### Infrasound array analysis, location and yield estimation

IMS infrasound array data within this study are analysed using the progressive multi-channel correlation (PMCC) method^15^ available from the DTK-GPMCC application in the National Data Center (NDC)-in-a-Box package. The main objective of the NDC-in-a-Box project and the interactive array processing tool DTK-GPMCC is to offer the capability of processing and analysing IMS waveform data to all NDCs of CTBT member states. PMCC is applied to the raw differential pressure recordings at each of the IMS infrasound arrays’ microbarometers to derive advanced data parameters like back-azimuth, apparent velocity and frequency content of coherent signals associated with different events. Back-azimuth reflects the horizontal direction of signal origin, while apparent velocity indicates the arrival inclination, where higher values correspond to propagation from higher-altitude ducts. Signals are identified as pixel information in distinct time steps and frequency bands, and they are clustered to signal families related to the same event. The third-octave band configuration with an inverse frequency-distributed window length is implemented^38^.

The PMCC method was applied to all IMS stations within 10,000 km distance. Signal parameters from five IMS infrasound arrays that could be associated with the Beirut explosion are provided in Table 1. Visualisation of the PMCC results for the three stations I48TN, I26DE and I17CI are provided in Fig. 2. Follow-up analyses of source localization and yield estimation using these PMCC results are provided in Fig. 3 and Table 2.

For quantifying the explosive yield using PMCC data, two different acoustic methods are established: The AFTAC relation (Air Force Technical Application Center)^18^ solely depends on the dominant signal period at maximum amplitude. The LANL relation (Los Alamos National Laboratory)^39^ depends on source-to-receiver distances and wind-corrected amplitude measurements, thus also incorporating climatological or real-time stratospheric wind profiles.

The source localization using the IMS infrasound network is based on a grid-search algorithm^8,40^. It relies on both the detected back-azimuths, the apparent velocities, and the arrival times at the stations. The grid covers the map shown in Fig. 3 with a resolution of 0.1$^{\circ }$. The detected back-azimuths are corrected by the atmospheric propagation conditions using the modelling method applied by Pilger et al. (2018)^41^, which also provides a celerity estimate based on the apparent velocity (the predicted celerity in Table 1 results from the mean PMCC values). For each grid point, the residuals of the corrected back-azimuths are computed and linearly weighted; the weight is one if the residual is zero, and zero if the residual is larger than the back-azimuth tolerance of 1$^{\circ }$ (for three stations) or 5$^{\circ }$ (for five stations). The tolerances are chosen in the order of the PMCC back-azimuth standard deviations. Also, the differential travel times of all two-station combinations are computed for each grid point and linearly weighted (the time tolerance is 90 s). The chosen time tolerance does not reflect the actual uncertainties resulting from the discrepancies between predicted and observed celerities (e. g., I17CI and I42PT; Table 1), which are due to atmospheric uncertainties in propagation modelling. The rather small tolerance ensures that only small differential travel times are accounted for. Consequently, the azimuth-based grid search is enhanced, as the back-azimuth is the more accurate parameter here. The sum of the weighted functions results in a two-dimensional probabilistic density function (PDF), the maximum of which is the optimum location of the grid-search algorithm.

Atmospheric profiles are assembled from high-resolution analysis fields (up to around 75 km) provided by the European Centre for Medium-Range Weather Forecasts (ECMWF) and the Horizontal Wind Model as of 2014 (HWM14)^16^.

**Table 1:** Parameters of the Beirut explosion signatures as derived from PMCC analysis of different IMS arrays.

Parameter	I48TN	I26DE	I17CI	I42PT	I11CV
Distance (km)	2455	2454	5130	5431	6240
Back-azimuth ($^{\circ }$)	88.4 (81.3–93.1)	126.5 (115.2–147.4)	47.2 (44.2–55.4)	77.9 (67.6–86.4)	60.7 (53.3–64.3)
Apparent speed (m/s)	357 (340–386)	350 (318–461)	341 (301–367)	341 (308–391)	355 (305–394)
Frequency (Hz)	2.57 (0.20–6.35)	0.70 (0.10–2.53)	0.37 (0.10–1.60)	0.66 (0.36–1.15)	0.84 (0.13–1.60)
Number of pixels (#)	7544	2802	944	62	58
Signal start (UTC)	17:06:17	17:11:19	19:44:06	20:20:42	20:44:56
Signal end (UTC)	17:27:34	17:28:46	19:57:35	20:24:02	20:56:17
Actual celerity (m/s)	294–347	291–333	296–310	287–290	299–309
Predicted celerity (m/s)	301	301	328	305	307

The mean and the range (in parentheses) are provided where parameters vary over pixels.

**Table 2:** Parameters and results for infrasound-based yield estimation using different IMS arrays and methods.

Parameter	I48TN	I26DE	I17CI	Resulting yield average
Dominant period at maximum amplitude (s)	4.6	4.6	5.4	
Maximum peak-to-peak amplitude (Pa)	0.48	0.12	0.13	
Stratospheric wind (HWM14) (m/s)	25.1	18.8	14.6	
Stratospheric wind (ECMWF) (m/s)	42.3	26.1	17.4	
Yield for AFTAC method (kt TNT)	0.86	0.86	1.47	1.06
Yield for LANL (+HWM14) method (kt TNT)	1.05	0.22	1.35	0.86
Yield for LANL (+ECMWF) method (kt TNT)	0.38	0.14	1.12	0.54

The effective wind speeds are averaged along the great-circle propagation paths between source and receivers.

### InSAR damage maps

**Figure 6:**
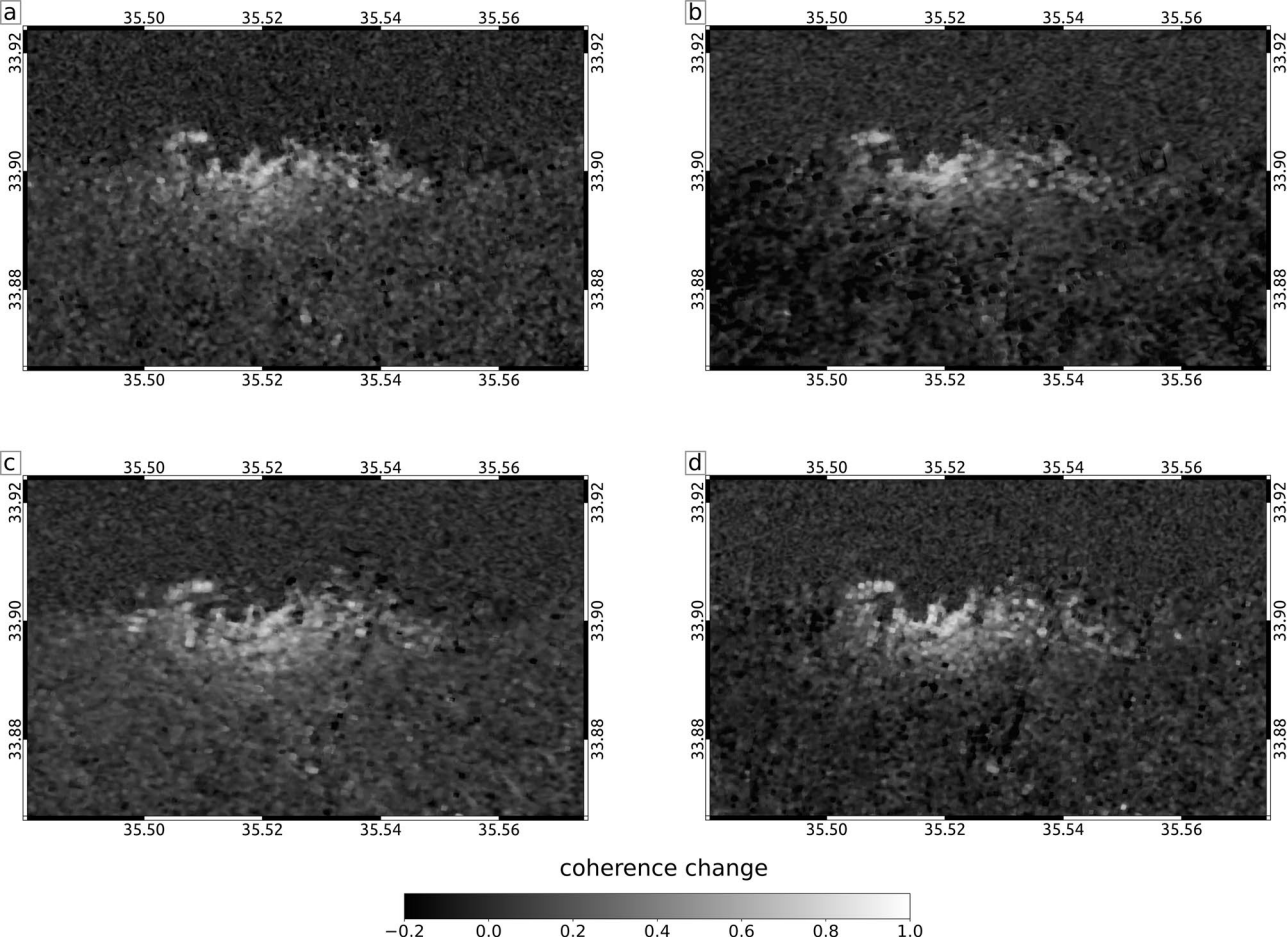
Sentinel-1 coherence change detection results for relative orbit 87 (**a**), 14 (**b**), 95 (**c**) and 21 (**d**).

Two ascending and two descending tracks of Sentinel-1 interferometric wide swath data pairs are used for the coherence change detection (CCD). For each track the acquisitions take place at different times and the radar waves also have different incident angles. Therefore, they sense the damage on the ground independently and from different observation geometries. Each track’s dataset consists of two acquisitions from before the explosion (pre-explosion) and a pair of acquisitions from before and after the explosion (co-explosion; see Table 3). The enhanced spectral diversity algorithm^42^ is used to precisely coregister the Sentinel-1 acquisition pairs on a burst level. Subsequently, the coherence is estimated for each acquisition pair using a 5 by 5 pixel window and an adaptive filter^43^. The coherence is a correlation coefficient of the complex SAR signal and ranges between 0 and 1. The results are geocoded and consist of eight coherence maps from four different acquisition geometries (two ascending and two descending). For each track we perform a CCD independently by subtracting the co-explosion coherence from the pre-explosion coherence (Fig. 6). The coherence loss of a single pixel is caused by e. g. damaged buildings or additive noise caused by a bias of the coherence estimator. By assuming that a coherence loss of $\ge$0.2 indicates damage, we create a binary representation of the coherence loss in form of damage and no-damage pixels. In order to increase the accuracy of the damage estimation, the four independent CCD maps are combined. If damage is indicated in at least one CCD map (CCD$\ge$0.2), we assign damage also in the combined binary damage image. How strongly an area is damaged is finally evaluated based on the percentage of damage pixels in a 10 by 10 pixel window (roughly 100 by 100 m), which forms the damage map (Fig. 4a).

**Table 3:** Details of the Sentinel-1 data used in the study.

Heading	Rel. orbit	Pre-explosion		Co-explosion	
		Primary	Secondary	Primary	Secondary
Ascending	87	29.07.2020	23.07.2020	04.08.2020	29.07.2020
Ascending	14	30.07.2020	24.07.2020	05.08.2020	30.07.2020
Descending	94	30.07.2020	24.07.2020	05.08.2020	30.07.2020
Descending	21	31.07.2020	25.07.2020	06.08.2020	31.07.2020

Data are acquired in interferometric wide swath mode by Terrain Observation with Progressive Scans (TOPS) in vertical/vertical (VV) polarization. The single look complex SAR images were downloaded from the Copernicus Open Access Hub.

### Yield estimation from InSAR damage maps

We link the InSAR observation to the yield via peak overpressure calculations, which give an expected level of damage for a certain yield at a given distance. We use the empirical relation “BOOM”^21^ for conventional explosive tests between 0.1 and 1 kt of TNT that relates the yield of an explosive to its resulting peak overpressure *P* (in kPa) at any given distance *r* (in m) for a surface explosion:

2 $$\begin{aligned} P = \frac{3.45978\times 10^{3} \cdot w^{0.444} \cdot A^{0.556}}{r^{1.333}} \end{aligned}$$

*A* is the atmospheric pressure (100.6 kPa, ECMWF data between 12 and 18 UTC) and *w* is the yield of the explosion in kt TNT equivalent. We note that some test settings under which “Yield estimation from InSAR damage maps” section Eq. 2 has been derived differ from settings present during the Beirut explosion, such as more effective explosives. Therefore, the yield-peak overpressure relation remains an approximation.

We assume the following relation between damage *D* and the peak overpressure *P*^22,44,45^ as given by “Yield estimation from InSAR damage maps” section Eq. 2 and we set 80 kPa peak overpressure to result in 100% damage to all structures:

3 $$\begin{aligned} D = \log _{10}(P(r)) \cdot \frac{100}{\log _{10}(80)} \end{aligned}$$

**Figure 7:**
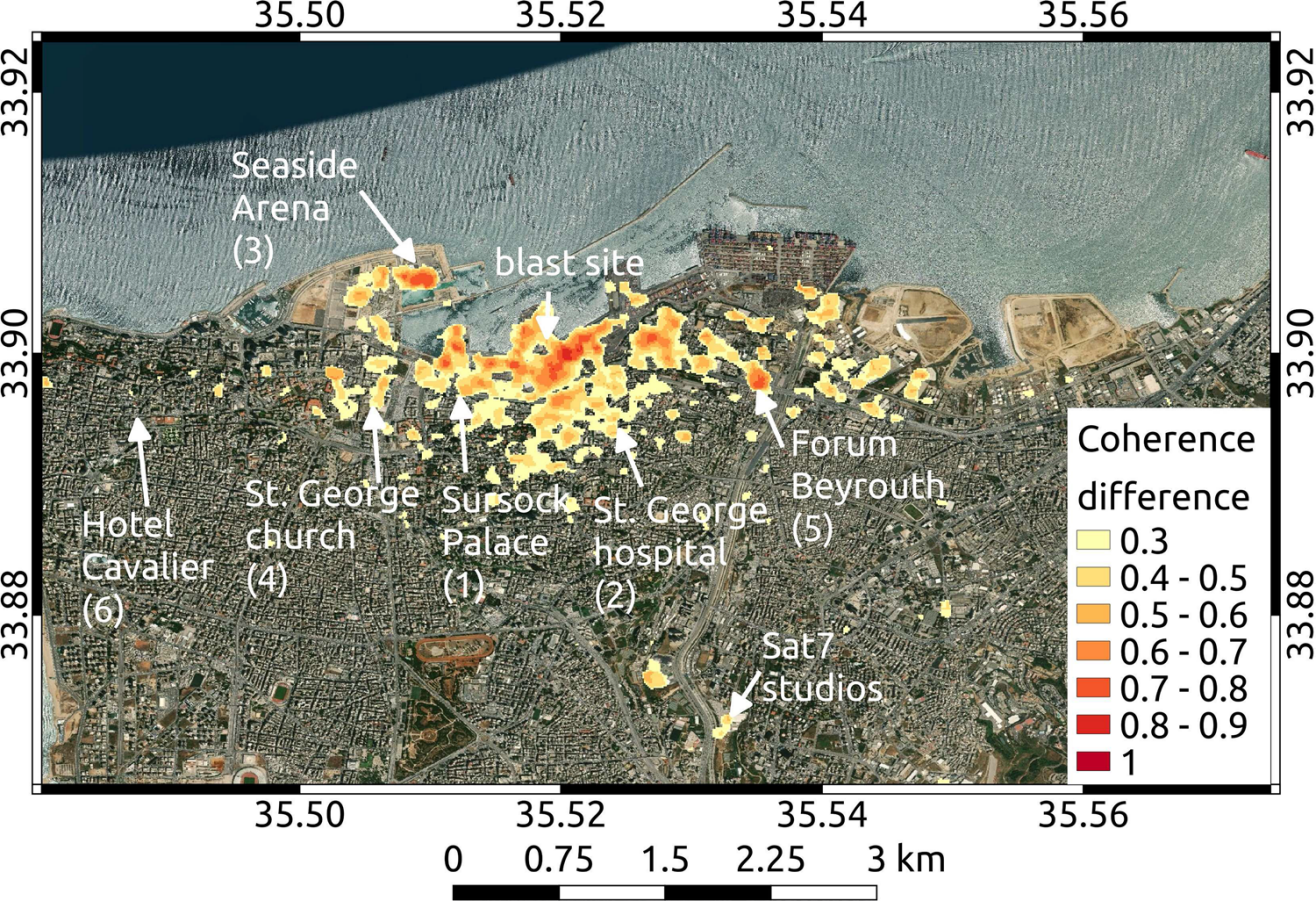
Low-pass filtered mean coherence loss map for illustration purposes. Shown is the coherence difference from all combined InSAR scenes. The color indicates the coherence difference, describing the coherence loss between scenes before and after the explosion. Coherence differences $<0.3$ are masked. Arrows point at notable locations and sites used as ground-truth (Table 3) as reference for calibration of “Yield estimation from InSAR damage maps” section Eq. 3. Map background was created using basemap data from the Esri ArcGIS Map Service.

The above equation is evaluated at the distances between the explosion and mapped damage pixels from InSAR data and solved for the necessary yield in TNT to cause the observed damage. We motivate our assumption of a common-logarithmic relationship between damage and peak overpressure by a similar empirical common-logarithmic relation between earthquake moment and earthquake damages. Such a relationship has been shown in e.g. macroseismic studies^46^ comparing values of the so-called Modified Mercalli intensity scale with earthquake magnitudes, which relate to the seismic moment with a common logarithm. For validation purposes we calculate the damage proxies from estimates of damage found in media reports (Fig. 7 and Table 4) and additionally use the open-source on-site inspection dataset^23,24^.

**Table 4:** Ground-truth used for validation of the relation between damage proxy inferred from InSAR data and the damage as function of peak-overpressure.

Number	Locality	Distance (km)	Est, damage	% of coherence loss (damage proxy)	Est. overpressure (kPA)	Computed damage proxy (%)
1	Sursock Palace	0.9	Partial demolition—partial collapse roof	59	7–15	44.4–61.8
2	Saint George hospital	1	Minor damage, partial demolition	47.9	8–15	47.5–61.8
3	Seaside Arena	1.1	Serious structural damage, collapse	61.5–74.3	18–40	66–84.1
4	Saint George church	1.4	Minor damage	58.9	7–15	44.4–61.8.75
5	Forum de Beyrouth	1.5	Metal buckled	52–60	7–15	46.3–61.8
6	Hotel Cavalier	3	Minor damage, buckling	None	3–7	28.26–36

See map of locations in Fig. 7. For each location the distance is given relative to explosion location and damage levels are estimated from available media coverage and reports^22,44,45^.

## Data Availability

Data from regional seismometers are available via FDSN services from GEOFON and IRIS. Data from global IMS infrasound arrays are available to National Data Centers of the CTBT and to others upon request through the virtual Data Exploitation Center (vDEC) of the IDC at https://www.ctbto.org/specials/vdec. Contains modified Copernicus Sentinel data 2020. SAR images used are openly available from the Copernicus Open Access Hub at https://scihub.copernicus.eu. We make the InSAR coherence maps and the inferred damage proxy map available on zenodo under doi: 10.5281/zenodo.4762436. ECMWF products, including the atmospheric model analysis, being no longer valid for forecasting are made available via https://www.ecmwf.int/en/forecasts/dataset (last accessed 21 Aug 2020) under CC-BY 4.0 License.
